# Therapeutic Relationship in eHealth—A Pilot Study of Similarities and Differences between the Online Program Priovi and Therapists Treating Borderline Personality Disorder

**DOI:** 10.3390/ijerph17176436

**Published:** 2020-09-03

**Authors:** Sandra Köhne, Ulrich Schweiger, Gitta A. Jacob, Diana Braakmann, Jan Philipp Klein, Stefan Borgwardt, Nele Assmann, Mirco Rogg, Anja Schaich, Eva Faßbinder

**Affiliations:** 1 Department of Psychiatry and Psychotherapy, University of Lübeck, 23562 Lübeck, Germany; ulrich.schweiger@helios-gesundheit.de (U.S.); diana.braakmann@uksh.de (D.B.); philipp.klein@uksh.de (J.P.K.); stefan.borgwardt@uksh.de (S.B.); nele.assmann@uksh.de (N.A.); mirco.rogg@uksh.de (M.R.); anja.schaich@uksh.de (A.S.); eva.fassbinder@uksh.de (E.F.); 2 GAIA AG Hamburg, 22085 Hamburg, Germany; gitta.jacob@gaia-group.com

**Keywords:** eHealth, borderline personality disorder, therapeutic alliance, schema therapy

## Abstract

eHealth programs have been found to be effective in treating many psychological conditions. Regarding Borderline Personality Disorder (BPD), few programs have been tested; nevertheless, results are promising. The therapeutic alliance is an important factor predicting treatment outcome in BPD. However, we do not know yet to what extent BPD patients form a therapeutic alliance with an eHealth tool and how this relationship differs from the relationship with their human therapist. This study aims to address this question using priovi, an interactive schema therapy-based eHealth tool for BPD. Semi-structured interviews were conducted to explore how patients perceived the therapeutic alliance with priovi and its differences compared to the alliance with their human therapist (N = 9). Interview data were analyzed following the procedures of qualitative content analysis. Additionally, the Working Alliance Inventory (WAI-SR) was administered in two versions (regarding the human therapist and priovi, N = 16) every three months during the treatment phase of one year. Results indicate that patients were able to form a good therapeutic relationship with priovi, but it differed from the relationship to their human therapist. Important categories were “priovi is helpful, supportive and always there” and “priovi is less flexible”. WAI ratings for the task subscale were high in both relationships but significantly higher in WAI_therapist_ compared to WAI_priovi_ in two measurements (nine-months measurement: t = 2.76, df = 15, *p* = 0.015; twelve-months measurement: t = 3.44, df = 15, *p* = 0.004). These results indicate that BPD patients can form a functioning alliance with an eHealth program and that eHealth programs may be especially useful for psychoeducation and cognitive exercises.

## 1. Introduction

Borderline Personality Disorder (BPD) is a prevalent [1] and complex mental illness associated with high utilization of mental health services [2,3] and serious impairments in psychosocial and occupational functioning [4,5,6], leading to high societal costs [7]. BPD can be effectively treated with specific psychotherapeutic methods. However, only few BPD patients receive these evidence-based treatments [8].

One way to provide more treatment facilities and to close the gap between demand and supply in the treatment of BPD is the implementation of internet interventions. In the past years, numerous eHealth interventions were successfully adapted for a broad range of mental disorders like depression, anxiety disorders, or post-traumatic stress disorder [9,10,11]. For BPD, research on internet interventions is also promising, but still in its infancy since most studies are still in early stages. One randomized controlled trial (N = 80) on an internet-based psychoeducation-tool for women with BPD [12] and several pilot studies on smartphone applications [13,14,15,16] showed to be efficient in reducing BPD or specifically targeted symptoms. These studies have also shown that patients perceived the apps as user-friendly and helpful [13,14,15]. A recent meta-analysis which included data from twelve studies of ten smartphone applications targeting BPD symptoms found no significant effect on reduction in BPD symptoms of those apps compared to face-to-face therapy or waitlist and concludes that more research is needed to determine if and how such apps could be implemented in providing mental health care for BPD [17].

The intervention used for the present study, priovi, is an internet intervention, based on schema therapy (ST) [18], which was specifically designed for BPD patients. A feasibility study of priovi in conjunction with individual face-to-face ST showed improvement in BPD symptoms in the participating patients. Also, qualitative data revealed that priovi was positively received by both patients and therapists [15]. A randomized controlled trial comparing priovi plus care as usual to care as usual alone is currently ongoing [19].

Priovi was developed by GAIA, a research and development enterprise in the field of e-health tools for mental disorders, together with clinical experts in BPD and BPD patients. It was designed to specifically address the needs and frequent mood shifts of BPD patients. As it is based on ST, priovi tries to establish a good therapeutic alliance with the patient by using a validating, soothing, and friendly tone. [20].

One of the reasons why there are so few studies on internet interventions of BPD are concerns that BPD patients need interpersonal contact and a stable therapeutic alliance in treatment and that these needs cannot be met by an e-health tool [20].

The therapeutic relationship is widely accepted as an important predictor of therapy outcome [21,22]. Also in patients with BPD, the therapeutic alliance has been found to be an important common factor predicting treatment outcome [23]. For internet-based psychotherapy, a meta-analysis found similarly strong associations between alliance and treatment outcome as in face-to-face psychotherapy [24]. It should be noted that most of the studies included in that meta-analysis targeted guided internet interventions, where an internet-based program is combined with regular support by a therapist and focused the alliance between patient and supporting therapist. Some studies also assessed the therapeutic relationship of patients with the online intervention itself and suggest that patients are in fact able to form a therapeutic alliance with a technology-based intervention [25,26,27,28,29]. One study implies, though, that this alliance may be different from the alliance to the therapist. Specifically, the affective bond seems to be lacking in comparison to the therapist, while there was no difference between the patients’ alliances with the online program and the therapists regarding the agreement on tasks and goals of the therapy [25]. Another study found patients could establish a stable working alliance with an avatar in an online intervention for insomnia, but some patients missed having a real therapist [26].

Until now we are only aware of quantitative studies to measure the alliance in internet interventions. However, to clarify the role of the alliance in internet interventions also qualitative interview studies are needed to understand how patients perceive this ‘technological therapeutic relationship’ and in what ways it differs from and is similar to a human relationship.

When it comes to BPD, we have no information (neither from quantitative nor from qualitative studies) to what extent patients can form a therapeutic alliance with an internet intervention and what the characteristics of such a relationship might be. This information will further our understanding of internet interventions in BPD. This information is crucial to improve and implement internet interventions for patients with BPD.

Thus, the present study aims to address the following research questions:

- To what extent are patients able to form a therapeutic relationship with priovi?
- What are the characteristics of this relationship?
- How does it differ from the working alliance with the human therapist

## 2. Materials and Methods

### 2.1. Recruitment and Sample

This study emerged from a pilot study concerning the feasibility and safety of priovi in conjunction with face-to-face ST at the Department of Psychiatry and Psychotherapy, University of Lübeck, Germany, in cooperation with GAIA AG (Hamburg, Germany), the company that developed priovi [15,20]. Approval was granted by the Ethics Committee of the University of Lübeck (AZ 14-038). All participants provided written informed consent for participation.

Inclusion criteria were a primary BPD diagnosis assessed via Structured Clinical Interview for DSM IV, Axis II (SCID II) [30] and a severity score above 20 on the Borderline Personality Disorder Severity Index (BPDSI) [31], willingness to participate in the study and written informed consent. For the qualitative interviews, patients also had to have worked at least halfway through priovi to ensure enough familiarity and encounters with the program. Exclusion criteria were acute psychosis and acute substance abuse.

Applying these criteria and including all participants who consented to the interview, it was possible to include nine patients for the qualitative interviews. For quantitative analysis, all 16 patients were included. The demographic characteristics of the sample are shown in Table 1. The mean BPDSI score at the beginning of treatment for this sample was 33 (SD = 6.7), mean age was 28.86 years (SD = 9.5, min = 20 years, max = 53 years). The nine patients participating in the qualitative interview had used priovi for 8.6 months on average at the time of their interviews (SD = 3.08, min = 5 month, max = 12 month). The mean BPDSI score for this subgroup was 34.37 (SD = 7.21), mean age was 32.78 (SD = 10.97, min = 21 years, max = 53 years).

### 2.2. Procedure

The semi-structured interview used for qualitative assessments in this study was developed by four of the authors (SK, EF, US, GJ). At the beginning of the interview, an explanation of the rationale for the qualitative study was provided for patients. The interview then started with an open question about priovi’s general significance to the patient followed by more specific questions about perceived similarities and differences between priovi and their therapist, as well as what was perceived as most helpful about both relationships. We also incorporated findings of two studies on working alliance in the interview: First, from a qualitative study examining what patients perceived as most healing about a therapeutic alliance [32] leading to questions regarding feeling “known as a person”, “get to the solution” and “relate to me”. Second, from a quantitative study which concluded that in face-to-face therapy unspecific elements (like the therapeutic alliance) might be especially effective, whereas in technological interventions specific elements (like psychoeducation and exercises) were more effective [25]. Regarding those findings, we asked participants whether or not they agreed with them to see if our sample was in accordance with earlier results on the topic or not.

The interview questions can be found in Appendix A. Participants were free to elaborate on these questions and a flexible interview style was adopted, in which questions could be skipped if they had already been answered by the participant beforehand and questions could be added if there were other aspects of both alliances patients perceived as important.

The interviews were conducted by SK and lasted between 35 and 69 min. The first round of interviews was held from February to June 2015. At that time, five patients were included based on inclusion and exclusion criteria. The second round of interviews was held from February 2016 to June 2017, including four more patients because they had progressed far enough within the program to participate. We allowed the patients to choose between conducting the interview via telephone or in-person, using office spaces at the university clinic, to allow for maximum comfort for the patient to encourage talking freely. Out of nine, six opted to conduct the interview via telephone, mostly because it was less time-consuming and more convenient, and three decided to do the interview in person. Interviews were recorded using audio recording devices and later transcribed.

For the quantitative assessments, patients filled out the German short version of the Working Alliance Inventory (WAI-SR) [33] every three months during the treatment period of one year, resulting in four assessments (T1–T4). The WAI captures three key alliance aspects within its subscales: agreement on “tasks” of therapy, agreement on “goals” of therapy, and development of an affective “bond” between therapist and patient. Each subscale consists of four items rated on a five-point Likert scale ranging from one to five. A total score and scores for the subscales can be calculated by summing the scores of the respective items divided by the number of items and ranges from one to five, with higher scores reflecting a higher quality of the alliance [33].

### 2.3. Data Analysis

Voice recordings of the interviews were transcribed verbatim by one of the authors (SK) and student research assistants, but did not reflect linguistic characteristics like pronunciation or length of pauses etc. The transcripts of the qualitative interviews were analyzed applying Qualitative Content Analysis [34], using QCA map (www.qcamap.org) for the initial categorization and later Microsoft Excel for further analysis. Two independent raters (SK and MR) were involved in the initial development of a preliminary category system. To derive this category system out of our data, an inductive approach was taken. First, three of the nine interviews were randomly selected. Both raters read these three interviews and identified meaningful passages according to the research questions. Those passages were then paraphrased and sorted; identical paraphrases in terms of content were summarized, meaningless paraphrases deleted. Paraphrases were then generalized and grouped by content until a first category system with a medium abstraction level emerged. The raters discussed their findings and then presented them to an expert group (DB, EF, US) where a consensual preliminary category system was created and used for further rating. After that, SK rated the rest of the interviews and differentiated the category system further, which was in turn also revised and finally consensually agreed upon within the expert group.

For quantitative analysis, means of the total scores of the WAI_therapist_ and the task subscale for both WAIs from the four assessments were described. Missing data were replaced using the last observation carried forward method. A total of 21.9% of data sets were missing. Of those, we replaced missing data sets where appropriate (71% of cases) and excluded participants from the particular analysis when no prior observation was available (29% of cases). The WAI subcategory ‘task’ was compared for all measurement states using paired t-tests to establish whether there was a significant difference between results.

### 2.4. Intervention

All patients received face-to-face ST sessions once a week for one year with their assigned therapist and a priovi account for one year to use according to their personal preferences. However, the therapist would encourage use and also frequently ask about their progress within the program as well as any experienced difficulties. Nine therapists took part in the study and treated between one and five patients each. Patients did not switch therapists unless organizationally necessary (e.g., when a therapist left the clinic).

Priovi is an ST-based interactive online tool, designed specifically for BPD patients. It is divided into two parts. The first part contains psychoeducation about BPD and ST in a conversational way. Patients can react to what priovi is explaining by selecting one of usually three to four predefined answers and priovi will then respond accordingly. All information is accompanied by further helpful and sometimes playful elements like audio messages or exercises (e.g., a safe place audio or pro/con lists for dysfunctional coping modes), comics, case examples, and illustrations to make the contents more tangible. Priovi has a protagonist, “Pia”, who is depicted in many of the comics and guides the user through the program. The second part is focused on specific exercises organized by mode (according to the ST mode model [35,36]), following a fixed order that reflects usual ST recommendations. During this phase, after the psychoeducation of phase one, the patient can work more extensively on his or her modes with a range of exercises which build up in difficulty. However, the patient can always skip parts that seem too difficult or that he or she doesn’t want to do (yet).

Priovi also provides some other features like a glossary explaining the most important terms around BPD and ST or a “mode toolbox” where exercises can be stored and revisited. A more detailed description of priovi can be found elsewhere [15,20].

## 3. Results

### 3.1. Qualitative Results

The qualitative analysis resulted in five key domains. Table 2 gives an overview of the derived category system with main categories and subcategories.

#### 3.1.1. Priovi Is Helpful, Supportive and Always There

Priovi’s big advantage as perceived by the patients was that it was always available, even when their therapist wasn’t. They stated that priovi was accessible around the clock and was also an unwavering source of comfort and validation that they could rely upon-priovi would never get frustrated and had unlimited time to explain, as many times as they wanted. They also reported that this helped them understand psychoeducational issues much faster. Priovi was also perceived as motivating because it encouraged patients and also reminded them to continue their work. Two patients reported priovi helped them to be more aware (e.g., of their current mode state) in their day-to-day life. One patient also mentioned priovi helped her feel much more self-efficient and self-reliant because she could work on her own and didn’t need her therapist every step of the way.

“I thought it was great that I could take it with me, use it on my cell phone. That was very valuable for me.”

*(priovi is always available, P4)*

“Priovi’s advantage is that it’s accessible anytime and that I can look things up anytime. I can do the exercises anytime. […] With my therapist I can only do that once or twice a week.”

*(priovi is always available, P4)*

“[Priovi’s validating communication style] means a lot to me. It’s a reason to keep working with the program. I think I would already have quit had it not been for that. I experienced enough lack of understanding from other human beings, I don’t need that electronically.”

*(priovi validates and normalizes, P5)*

“I can just work more independently with it than I could with just face-to-face therapy. This makes me feel a whole lot more self-effective and less reliant; if something doesn’t work out, I don’t have to wait until I can discuss it in therapy, I can do something myself.”

*(priovi promotes self-efficacy, P9)*

#### 3.1.2. Priovi Evokes Emotions (But Not as Much as a Human)

Patients reported that the work with priovi elicited a range of emotions, just like one would expect of a human relationship. One patient initially reported a very positive attitude towards priovi, which she viewed almost like a friend, and then a rupture in the relationship after she felt abandoned and misunderstood by the program due to technical errors (e.g., text messages stopped coming). Several patients felt priovi evoked emotions (e.g., via its exercises) but then left them alone in dealing with those emotions. Especially the daily text messages were mentioned to elicit many pleasant emotions because it was seen as priovi getting in touch with the patients, without them having to do anything for it. Five patients also reported several aversive emotions when working with priovi, some were annoyed at times (e.g., because the communication style was viewed as “too soft” and childlike or because it felt like a chore) or that sometimes priovi’s messages would make them feel bad. One patient also stated priovi reminded her of her disorder. Patients made clear, however, that a human counterpart always elicited more emotions than priovi could. But this also held some advantages for the patients—several of them reported being able to work more stringently with priovi, especially on psychoeducation and cognitive tasks, than with their therapist because beyond evoking less emotional ups and downs, they were not afraid of being judged by priovi. This is reflected by the subcategories “priovi can’t judge” in this category, as well as by “priovi is always the same”, further explained within the category “priovi is less flexible”.

“In a personal relationship, the judging part of my brain is more active [than in the relationship with priovi], because the program can’t devalue me. Not really. Not even I think “Priovi thinks I’m crap”. My mind can’t go there with priovi, but in a human relationship, it can.”

*(priovi can’t judge, P6)*

“I’m a bit anxious about the program because I know that once I’m in an emotional spiral, I can’t get out of it quickly by myself.”

*(priovi leaves me alone with my emotions, P7)*

“[…] kind text messages like ‘Have you already done something nice for yourself today?’ or ‘It’s great that you’re here’ or something like that, that’s like the sun shining on you… it just does tremendous good, to have something like that right in the middle of your busy day-to-day life, and it’s not only in that moment, it’s something to carry with you over the next couple of hours.”

*(priovi evokes pleasant emotions, P8)*

“From one day to the next, I stopped getting any more text messages and that made me really fall apart because I thought I could rely upon getting one every day, and then suddenly I got nothing.”

*(priovi’s technical errors evoke emotional pain, P8)*

#### 3.1.3. Priovi Is Less Flexible

Especially in comparison with their human therapist, patients found priovi to be less flexible, as it was less able to respond to their individual problems or current emotions. Also, they stated that their therapist knew their biography which made a big difference for many patients—they felt this enabled their therapists to better understand what was going on with them. Many patients felt priovi covered the basics of BPD and ST well, but for any specific question, they still needed their therapist. Several patients mentioned that their therapist could correct them when they had gotten something wrong whereas priovi couldn’t. They also noted that priovi couldn’t change, it could only offer what was preprogrammed and if they didn’t like some part of it, there was no flexibility to adapt it. However, a positive aspect of this rigidity, reported by two patients, was that it also offered stability-priovi’s explanations and exercises never changed, which made it easier for them to rely upon them because they regarded it as “pure” information without the possibility of being clouded by a human’s judgment for example. They also felt that with priovi they knew what was coming, whereas a therapy session could always go in many different directions.

“I feel like I can stabilize what I’ve learned more cleanly with priovi because I always read the same explanations, I always do the same exercises. I feel this way there are fewer steps in between. With a human counterpart, there is always that other person’s judgment or impression that factors into it, and with priovi, there isn’t.”

*(priovi is always the same, P3)*

“It’s just more personal with a human counterpart who can directly answer any question I have, which may not be included in the program. I think I need that human counterpart to learn how to trust and just to talk about things and ask things that the program can’t answer for me.”

*(priovi isn’t very flexible or individual, P9)*

“Well, [the therapist] is just more flexible. And it may be easier for someone knowing my biography to identify triggers for current moods.”

*(priovi isn’t very flexible or individual, P7)*

“Well, [priovi] works with general hypotheses, which are surely correct but also too general for me.”

*(priovi is general, P5)*

“Priovi is like a box, it’s got its content but nothing more can fit in. But my therapist doesn’t stand still, there is always something new, there’s food for thought and new reactions. Priovi can’t think. It’s got its content and that’s it.”

*(priovi isn’t very flexible or individual, P6)*

#### 3.1.4. There Are Links between the Relationship to the Therapist and Priovi

Some patients reported that the two relationships with their therapist and priovi affected one another in certain ways. Two patients reported that a good relationship to the therapist who had recommended priovi helped them get over initial fears of using priovi - since they trusted their therapist, they also trusted their recommendation to use priovi. One patient mentioned that the good relationship to her therapist motivated her to use priovi more because she wanted to please her therapist and also trusted her opinion of the program. Another patient reported the work with priovi had even strengthened her bond to the therapist because priovi allowed her to work continuously on her issues which improved quickly and she felt more self-efficient.

“Well, what was also part of it was that I thought ‘my therapist will be glad and she thinks it’s good for me to do this’ and then I also knew it really does help me so I thought ‘I’ll just do my exercises’.”

*(good relationship to therapist motivates use of priovi, P4)*

“Since I have such a close, trusting relationship with my therapist and really trust her completely, I knew this [priovi] can’t be bad, it’ll be okay if she says so. I just knew I could rely upon that.”

*(good relationship to therapist prevents fear of using priovi, P9)*

“If I hadn’t worked so continuously on so many things with priovi, the trusting relationship to my therapist wouldn’t have gotten that much closer, as it did. It strengthened and improved it because I just worked continuously on this all and I realized I was making progress, I was feeling self-efficient. And that deepened the bond to my therapist.”

*(working with priovi strengthens bond to therapist, P9)*

#### 3.1.5. Priovi Is No Human

All the patients in our sample reported they felt a profound difference between their relationships to priovi and the therapist because priovi “just isn’t human”. Even if they were quite satisfied with priovi and even personified it to a certain extent, they still felt a significant difference compared to a human relationship. However, many patients could identify to a certain degree with the program, felt somewhat seen as a person by it and some even saw priovi almost as a person who understood them and who they could talk to. Most patients, however, saw priovi as an electronic tool, without many human traits, that mainly provided information and helped them understand BPD, ST, and subsequently themselves, better. They stressed this cognitive component much more than any emotional one, which many said was rather dealt with in their human relationship. One patient mentioned priovi helped her explain her illness to significant others, which led to less stigmatization due to the BPD diagnosis in her life.

“[…] it really felt like having a friend, like I’d met Pia [Priovi’s protagonist] and when I had a problem I wondered if maybe there was something in priovi about it and I looked it up.”

*(Priovi feels almost like a person, P4)*

“Right in the beginning, I was able to choose my nickname [as the name priovi would use to address me]. That, and the text messages I got, always kept me from feeling treated like ‘just a number’, neither by priovi nor by my therapist.”

*(It is possible to identify with priovi, P2)*

“The difference is an emotional one. Electronics don’t have emotions. They don’t have brains. And therefore no human traits.”

*(priovi is less emotional than a human, P5)*

“I could take the program to my family, who kind of frowned upon my borderline diagnosis and didn’t really know what it was, and then I could show them on the screen and they could read about it and they understood it. That made things much easier.”

*(priovi helps explaining BPD to others, P4)*

“To me, it’s like several modules that you work through to attain a certain level of know-how, mostly on a cognitive level. Face-to-face therapy operates much more on an emotional level.”

*(priovi provides information, helps to understand and priovi is less emotional than a human, P7)*

### 3.2. Quantitative Results

Table 3 gives an overview of mean scores for the total score of WAI_therapist_ and the task subscale for both WAIs during all four measurement states. Please note that in the WAI_priovi_ only six items were assessed and thus the total scores and the scores for the subscales for bond and goal were not calculated. For the task subscale, all items were recorded in both WAIs, and paired t-tests were carried out to check for significant differences between the two. Results for the first measurement state (after 3 months of treatment) indicate no significant difference between the two WAIs (t = 1.48, df = 11, *p* = 0.166). The second measurement state did not yield any significant results either (t = 1.54, df = 14, *p* = 0.146). The third and fourth measurements (after 9 and 12 months of treatment, respectively) both show significant differences with higher ratings for the WAI_therapist_ (third: t = 2.76, df = 15, *p* = 0.015; fourth: t = 3.52, df = 15, *p* = 0.003) with medium to strong effects (Cohen’s d = 0.69 for third measurement and 0.88 for fourth measurement).

## 4. Discussion

We investigated the relationship between patients with BPD and the online program priovi on a quantitative and qualitative level and compared it to the relationship with the therapist providing face-to-face ST. To our knowledge, this is the first study to explore the working alliance with an internet intervention for BPD patients. Results show that patients were able to form a relationship with priovi but that it significantly differs from a human relationship. The quantitative results indicate that patients were more in agreement with their therapist about what tasks should be accomplished in therapy than with priovi during the second half of treatment. The qualitative results also indicate significant differences between the two forms of working alliance. In short, patients appreciated that priovi was always available and provided psychoeducational content and cognitive exercises, that helped them to progress in therapy. Although all patients felt validated by priovi and most could identify with priovi to a degree and some even felt it was almost like a relationship with a real person, the relationship with their therapist was perceived as being emotional on a deeper level and more individually suited to their needs.

These findings are in line with earlier qualitative pilot studies on how BPD patients experience online interventions, which report good acceptance, satisfaction, and usability of online tools [13,14,15] and good WAI ratings for technological interventions [25,26,27,28].

The WAI ratings of this study are comparable to those of outpatients in the study of Munder et al. [33]. A study with insomnia patients about the therapeutic relationship to an avatar found that the WAI subscale goal was rated lower for the avatar while task results were comparable to a human relationship [26]. Ratings of the WAI_priovi_ for the task subscale in our sample (M = 3.54, SD = 0.86, after three months of treatment) are comparable to those of a study by Heim et al. [27] (M = 3.24, SD = 0.79, after three weeks of treatment), although ratings for human therapists were significantly higher at two measurement states in our sample.

Berger et al. [27] report a mean WAI task score of 3.8 (SD = 0.58) and 3.23 (SD = 0.69) for their tailored (to the specific anxiety symptoms of the patient) and untailored conditions respectively; scores for the WAI_priovi_ in this study are in between those results, although priovi is also highly tailored for BPD patients.

A narrative review by Berger [29] also finds alliance ratings for internet interventions to be roughly equivalent to those measured in face-to-face therapy, irrespective of communication format, diagnostic group, or amount of contact to the therapist. It also points out that the unique characteristics of the alliance in different therapeutic formats need further study.

An alliance-outcome association for the task and goal subscales was found by Penedo et al. [28]; Heim and colleagues [26] report that results of the WAI subscales were not associated with outcome in their trial, however, the affective bond with the avatar and whether or not patients missed a human therapist were. The authors noted that a human therapist was increasingly missed as treatment progressed, which they attributed to the use of later stages in the app: similar to priovi, it delivers mostly psychoeducation in the beginning and more specific content in the form of a (restricted) dialogue later on. The authors conclude that the increased desire for a human therapist is probably due to the restricted form of dialogue the app can offer, which would be in line with one of the main findings of this study, “priovi is less flexible”.

It is also interesting to note that we found significant differences between the WAI_therapist_ and WAI_priovi_ only in the two last measurement states; examination of the mean task scores indicates that WAI_priovi_ scores are higher during the first two assessments and decrease somewhat after that whereas the WAI_therapist_ scores seem more stable over time. This might be attributable to priovi being especially valuable during the phase of treatment where psychoeducation and learning of new skills are most prominent. Several patients reported this during the interviews and also mentioned having used priovi less later in treatment when they were already able to cope better on their own, and thus its relevance would also decrease over time. However, some patients also reported greater difficulty using priovi during phase two, as the program shifts from mainly delivering psychoeducation to exercises, which may also contribute to less usage.

Patients clearly stated that although priovi evokes emotions, these emotions are not as intensive as with their therapist. While the emotional connection is often regarded as a main healing factor in a human therapeutic alliance, for some patients priovi’s lower emotional impact was perceived as an advantage because they could concentrate better and were able to work more easily with priovi on exercises and psychoeducation than in personal sessions. During face-to-face therapy, these patients were usually so preoccupied with keeping up with the interaction and their thoughts and feelings about it, that they had a hard time processing information and concentrating on any exercise. This is an important aspect for the treatment of BPD patients with high emotional dysregulation and interpersonal problems indicating that teaching psychoeducational and cognitive content might be especially suitable to be done by an internet intervention. This could enhance and speed up treatment effects and disburden therapists if a combination of online treatment and face-to-face psychotherapy is offered.

Priovi’s most prominent advantages, which were mentioned by nearly all participants, are its availability and its unwaveringly validating style of communication. Priovi offers information and comfort 24/7. It never gets frustrated, tired, or angry. Patients can also take as much time as they need to complete exercises or read explanations whereas therapy sessions are always time-limited. Being able to repeat psychoeducation and exercises in between sessions was perceived positively by patients. They felt enabled to learn more quickly, sometimes to improve faster and to feel more self-efficient. This level of stability and availability is not possible to attain for a human therapist, and thus, might be a huge advantage of online interventions

On the other side, Priovi’s most clear disadvantage is its inability to react to patients more flexibly and individually. This was observed by all patients in our sample. Patients found priovi to be less able to respond to their needs in specific situations or when they were in difficult emotional states. Patients felt the need to talk personally about their issues with their therapist. They couldn’t ask priovi specific questions spontaneously and felt priovi was providing a general model of BPD and ST but that they needed their therapist to create their individual model of the disorder and understand their personal and unique issues. This is of course an inherent limitation of all technological interventions in this field.

Interestingly, one patient who initially reported a very favorable view of priovi, reported a rupture in her relationship to priovi as technological errors in the system arose (e.g., text messages stopped coming) which changed her attitude to the program completely. Patterns like this (idealization/devaluation) can also be observed in many human relationships with BPD patients; however, online interventions are not able to repair these ruptures. This implies that eHealth interventions are good additions to traditional therapy but probably not very well-suited to be used as stand-alone tools in many cases, especially in severe and complex BPD patients. This is in line with existing data on this topic [37]. However, if no traditional therapy is available, priovi could still offer some psychoeducation and comfort, as it has been demonstrated that internet-based psychoeducation can reduce BPD symptoms and improve social functioning [12]. Moreover, internet interventions have been shown to be suitable low-threshold interventions, also for patients with a high symptom burden [38]. Priovi seems to be well suited for cognitive exercises and delivering psychoeducation but cannot meet individual needs or address personal issues.

While the therapeutic relationship has for the longest time been considered an underlying, facilitating factor for change, regardless of the psychotherapeutic method used, there have recently been some researchers questioning this view. Schweiger et al. [39] suggest that the therapeutic relationship may be viewed as an emergent phenomenon, resulting not only from “chemistry” between therapist and patient but is composed by different aspects interacting with each other such as professionalism, emotional bond, plausible models, explanations and suitable techniques, or experience of change early in treatment. Given our results, it might be that specific ingredients of the therapeutic alliance can be better addressed by a human therapist (e.g., emotional bond, corrective experience of a healing relationship or a new way to deal with emotions and needs) while others might be sufficiently or even better addressed by online interventions (e.g., explanation of plausible models, psychoeducational content or explanation of specific techniques).

We did not measure alliance-outcome associations. Literature shows mixed results in this regard. Patterns of findings suggest that it is rather the agreement on goals and tasks than the personal bond that is important for outcome in internet interventions [40]. Thus, the therapeutic alliance in internet interventions might operate differently, and finally, one cannot clearly state that it is as important as in face-to-face psychotherapy. Heim et al. [26] suggest that the use of an avatar may facilitate the formation of a relationship, which could also be true for priovi, as it has a protagonist, Pia, to whom patients may connect on a more personal level than to text-only interventions.

This study has several limitations. Most prominently, the small sample size is a major limiting factor. Furthermore, the low number of male participants (two for quantitative results, none for qualitative results) restricts what these results can tell us about online tools for male BPD patients. The WAI_priovi_ is not a validated, reliable psychometric instrument, but was modified from the WAI-SR (since validated measures were lacking at the time the study was designed) as a first exploration of the working alliance in an online intervention for BPD.

Moreover, in this study priovi was offered in conjunction with weekly face-to-face sessions with ST. We cannot be sure if patients developed a relationship to priovi per se or rather to the concept of ST which it symbolizes. It would probably be reasonable to assume that both priovi as an interactive “face” of ST and ST as a concept are involved in this kind of relationship and are hard to separate.

Possible applications of internet interventions like priovi may be low-threshold delivery of psychoeducation, cognitive content and validation as well as using it in addition to traditional face-to-face therapy to speed up learning and acquisition of new skills along with having a reliable source of comfort outside of therapy sessions.

Future research should include larger sample sizes and more male participants as well as a reliable and valid instrument to capture alliance in Internet Interventions such as the newly developed Working Alliance Inventory for Internet Intervention (WAI-I) [41]. Also, more qualitative research to better understand the specific characteristics of the therapeutic relationship with the online intervention and with human therapists is urgently needed.

## 5. Conclusions

In conclusion, this study shows that BPD patients were able to form a relationship with the online tool priovi but that this relationship differed significantly from their relationship to the therapist. This difference seems to be mainly emotional, meaning that priovi elicits fewer emotions which made working with it less emotionally intense. Thus, priovi is less suitable for treating interpersonal or emotional issues but enabled some patients to be more focused and calmer during cognitive exercises and psychoeducation. Priovi’s main strengths are that it is always available, can offer an unlimited amount of validation and comfort at any time, and can help to understand psychoeducational and cognitive contents quickly. Priovi’s main disadvantages compared to a human therapist are its limited flexibility and individuality.

The results of our study could inform further development and implementation of online interventions in the treatment of BPD. Future online interventions could carefully take into account the above-named limitations, but also display the strengths reported by our patients.

## Figures and Tables

**Table 1:** Demographical and clinical characteristics of the sample (N = 16).

Characteristic	N	%
**Gender**		
Male	2	12.5
Female	14	87.5
**Highest Education**		
Secondary school (9 years)	1	6.25
Secondary school (12/13 years)	4	25
Vocational training	6	37.5
University degree	5	31.25
**Employment Status**		
Student/apprentice	2	12.5
Voluntary service	1	6.25
Employed	8	50
Unemployed	2	12.5
Incapacitated for work	3	18.75
**Comorbid Psychiatric Disorders (DSM IV)**		
Axis I		
Affective disorders	13	81.25
Substance use disorders	6	37.5
Anxiety disorders	12	75
Obsessive-compulsive disorder	3	18.75
Posttraumatic stress disorder	11	68.75
Eating disorders	9	56.25
Axis II (except BPD)		
Obsessive-compulsive personality disorder	5	31.25
Schizotypal personality disorder	1	6.25
Avoidant personality disorder	5	31.25
Paranoid personality disorder	3	18.75
Narcissistic personality disorder	1	6.25

**Table 2:** Category System: Facets of a relationship to priovi.

Category	Subcategory	N	%
Priovi is helpful, supportive and always there	Priovi is always available	8	88
	Priovi validates and normalizes	9	100
	Priovi motivates	3	33
	Priovi promotes self-efficacy	1	11
	Priovi helps being more aware	2	22
Priovi evokes emotions (but not as much as a human)	Priovi can’t judge	3	33
	Priovi leaves me alone with my emotions	4	44
	Priovi’s technical errors evoke emotional pain	1	11
	Priovi evokes pleasant emotions	5	55
	Priovi evokes aversive emotions	5	55
Priovi is less flexible	Priovi isn’t very individual or flexible	9	100
	Priovi is general	7	77
	Priovi is always the same	2	22
There are links between the relationship to priovi and the therapist	Good relationship to therapist prevents fear of using priovi	2	22
	Good relationship to therapist motivates to use priovi	1	11
	Working with priovi strengthens bond to therapist	1	11
Priovi is no human	Priovi is less emotional than a human	9	100
	Priovi feels almost like a person	4	44
	It is possible to identify with priovi	2	22
	Priovi provides information, helps to understand	6	66
	Priovi helps explaining BPD to others	1	11

**Table 3:** Overview of mean scores for WAI_therapist_ and WAI_priovi_ for four measurement states.

Measurement	M	SD	N
**T1**			
WAI_therapist_ total	3.97	0.47	13
WAI_therapist_ task	3.83	0.61	13
WAI_priovi_ task	3.54	0.86	12
**T2**			
WAI_therapist_ total	3.89	0.73	16
WAI_therapist_ task	3.73	0.79	16
WAI_priovi_ task	3.47	0.73	15
**T3**			
WAI_therapist_ total	4.04	0.53	16
WAI_therapist_ task	3.83	0.68	16
WAI_priovi_ task	3.33	0.83	16
**T4**			
WAI_therapist_ total	4.11	0.57	16
WAI_therapist_ task	3.98	0.73	16
WAI_priovi_ task	3.45	0.82	16

Note: M = mean, SD = standard deviation, T1 = measurement state 1 (after 3 month of treatment), T2 = measurement state 2 (after 6 month of treatment), T3 = measurement state 3 (after 9 month of treatment), T4 = measurement state 4 (after one year of treatment).

## Appendix A. Semi-Structured Interview: Therapeutic Relationship to Priovi/Therapist

Introduction: Thank you for taking the time to do this interview today! I want to understand the kind of relationship you developed with priovi. This may sound strange, as priovi is a program, but some studies have shown that people can in fact form certain kinds of relationships with objects or programs. So I would like to ask you to share your thoughts about priovi and what priovi means to you.

- What does priovi mean to you?
- Are there any other programs or objects which have a similar significance to you? If so, what are similarities and differences compared to priovi?
- What does your therapist mean to you and how does it compare to what priovi means to you?
- How much do you feel priovi is embedded in your face-to-face therapy?
- How much do you feel priovi is associated with your therapist?
- Studies suggest that in order to have a successful therapeutic relationship, it is necessary to feel “known as a person”. What do you think about that? Are there any differences between priovi and your therapist regarding this?
- (also ask this question for other categories: „get to the solution“ and „relate to me“) [32]
- Studies also suggest that technological interventions such as priovi mainly help via specific elements like psychoeducation or exercises whereas traditional therapy mainly helps via unspecific things like the therapeutic relationship. What do you think about that? [25]
- What do you feel helped you most about priovi/traditional therapy?
